# Industrial Accidents in War-Time

**Published:** 1918-06-15

**Authors:** 


### INDUSTRIAL ACCIDENTS IN WAR-TIME.

The factors concerned in the causation of industrial accidents, those unforeseen and fortuitous injuries which befall workers, have been made the subject of investigation by Dr. H. M. Vernon for the Health of Munition Workers Committee, and the results have been published in the Committee's Memorandum No. 21. Happenings which are properly classed as accidents have always been regarded as partaking in some degree of the mysterious and have been attributed, according to prevalent superstition, to such influences as evil spirits, the visitation of God, and ill-luck. Industrial accidents have the quality of being divisible into those in which acts of the worker are, or may be, conducive to the accident and those in which they are not. The report under consideration, which the Committee considers to be the more valuable in that so far as they are aware no similar inquiry has been undertaken, recognises the importance of the practical application of the results of the investigation to the prevention or reduction in number of accidents. Such obvious considerations as defects of machinery and the absence of machinery guards have been excluded, but most of the other factors in accident causation are referred to in the report.

The conclusions arrived at reveal no striking novelty: " The majority, probably, of industrial accidents are unavoidable; at best one can only hope to reduce their number, and never to eliminate them entirely." " Increased speed of production inevitably tends to a more than proportional increase of accidents." " Accidents depend, in the main, on carelessness and lack of attention of the workers." The ideal condition suggested for the worker is put forward with great diffidence, and it must be agreed that the imaginary picture of an isolated munition worker, enjoined to silence, with ears plugged to shut out machinery and other noises, and partitioned off in solitary confinement, is not an attractive one from the worker's point of view, however conducive such a state might be to a condition of " mental calm and equilibrium " !

" The careless habit of mind can be diminished by stricter sobriety. There can be no doubt that the less alcohol the worker consumes the better it is for the quality and quantity of his work, and for his accident immunity." This is true, but it is also true that fatigue and other " psychical influences " may, too, produce carelessness and inattention; " the psychical factor is one of the most important in accident causation." The same " careless habit of mind " may be contributory both to indulgence in alcohol and to liability to accident, and the evidence obtained during the investigation of any direct present-day connection between alcoholic drink and the occurrence of accidents appears to be scanty.

The report is founded upon a very earnest and painstaking inquiry, and forms a useful and natural corollary to the memoranda of the Committee on hours of work and industrial fatigue, in conjunction with which it should be widely read.

